# Serum amyloid A is a better predictive biomarker of mucosal healing than C-reactive protein in ulcerative colitis in clinical remission

**DOI:** 10.1186/s12876-020-01229-8

**Published:** 2020-04-03

**Authors:** Masaki Wakai, Ryohei Hayashi, Shinji Tanaka, Toshikatsu Naito, Junko Kumada, Motonobu Nomura, Hidehiko Takigawa, Shiro Oka, Yoshitaka Ueno, Masanori Ito, Kazuaki Chayama

**Affiliations:** 1 Department of Gastroenterology and Metabolism, Hiroshima University Hospital, 1-2-3 Kasumi, Minami-ku, Hiroshima, 734-8551 Japan; 2 Department of Endoscopy, Hiroshima University Hospital, 1-2-3 Kasumi, Minami-ku, Hiroshima, 734-8551 Japan

**Keywords:** Mucosal healing, Serum amyloid a, Ulcerative colitis, C-reactive protein

## Abstract

**Background:**

Many studies have revealed that mucosal healing improves the long-term prognosis of ulcerative colitis. Frequent colonoscopy is difficult because of its invasiveness and cost. Therefore, in diagnosing and treating ulcerative colitis, noninvasive, low-cost methods for predicting mucosal healing using useful biomarkers are required in the clinical setting.

This study aimed to evaluate whether serum amyloid A is a better serum biomarker than C-reactive protein in predicting mucosal healing in ulcerative colitis patients in clinical remission.

**Methods:**

Ulcerative colitis patients whose C-reactive protein and serum amyloid A were measured within 1 month before and after colonoscopy were included in this retrospective study, and the relationship between the C-reactive protein and serum amyloid A values and the mucosal condition was analyzed. Mucosal condition was assessed using the Mayo Endoscopic Score, with score 0 or 1 indicating mucosal healing.

**Results:**

A total of 199 colonoscopic examinations were conducted in 108 ulcerative colitis patients who underwent C-reactive protein and serum amyloid A blood tests. In clinical remission patients, serum amyloid A showed a strong correlation with mucosal inflammation compared to C-reactive protein and had excellent sensitivity and specificity rates with significant statistical significance.

**Conclusions:**

Serum amyloid A is a more useful marker compared to C-reactive protein in predicting mucosal inflammation in ulcerative colitis patients in clinical remission.

## Background

Patients with ulcerative colitis (UC) typically manifest symptoms such as rectal bleeding, persistent bloody diarrhea, increased stool frequency, and abdominal pain [1, 2]. Moreover, UC is a chronic inflammatory disorder of the colorectum characterized by repeated clinical remission and relapse [3]. UC is generally thought to be caused by various factors, such as genetic factors, immune abnormality, and environmental factors including intestinal bacteria [1]. However, its cause has not been elucidated yet, and its fundamental therapy has not yet been established. The goal of UC treatment is to achieve clinical remission in patients; however, recently, treatment options, such as immunomodulators and biological agents, have been gaining attention, and the therapeutic goal has changed from achieving clinical remission to achieving mucosal healing [1].

Mucosal healing is related to long-term clinical remission, and long-term prognosis is improved by reducing the risk of hospitalization and surgical operation [4]. Mucosal healing is detected via endoscopy, as colonoscopy is quite an invasive examination, and frequent examinations are difficult because of its medical cost. Therefore, in the diagnosis and treatment of UC, noninvasive, low-cost prediction methods of mucosal healing using useful biomarkers are clinically required. Generally, C-reactive protein (CRP) is reported to be less sensitive in UC cases [5], and it mildly increases in UC than in Crohn’s disease (CD). Although serum amyloid A protein (SAA) is mainly secreted from the liver, similar to CRP [6, 7], SAA is reported to be more effective than CRP in diseases other than inflammatory bowel disease (IBD) [8]. In addition, SAA was reportedly correlated with the clinical activity in UC [9, 10], but there is no report examining its correlation with endoscopic findings. Therefore, this study aimed to evaluate whether SAA is a better serum biomarker than CRP in predicting mucosal healing in UC patients in clinical remission.

## Methods

### Patients

This study included consecutive outpatients or inpatients who underwent endoscopic examinations at Hiroshima University from April 2010 to March 2017. CRP and SAA values of these patients measured within 1 month before and after colonoscopy were retrospectively analyzed. The exclusion criteria were as follows: patients who were administered new therapies during the period from colonoscopy to the time when CRP and SAA measurements were taken, those with other infections such as common cold, those with concurrent autoimmune diseases such as collagen diseases, and those who were not in clinical remission. UC diagnosis was made based on the clinical, endoscopic, and pathological findings. Demographic, clinical, endoscopic, and laboratory data were obtained from patients’ medical records. Clinical symptoms were evaluated using the Rachmilewitz clinical activity index (CAI). Self-exclusive symptoms (weekly frequency of bowel movement, bloody stools, and abdominal pain), objective symptoms (temperature and investigator’s assessment of symptomatic state), extraintestinal manifestations, and blood test findings (sedimentation rate and hemoglobin) were divided into seven items and evaluated using the total score (range: 0 to 29). Clinical remission was defined as a CAI of 4 or less [11]. In addition, the Montreal classification was used to define the extent of the lesion, which was classified into three types: ulcerative proctitis (E1), left-sided UC (E2), and extensive UC (E3) [12].

### Evaluation via endoscopic examination

Mayo endoscopic subscore (MES) was used to evaluate the degree of mucosal inflammation in each part of the colorectum (cecum, ascending, transverse, descending, sigmoid colon, and rectum). Mucosal inflammation was analyzed using the maximum value among the scores. Mucosal healing was defined as MES 0 or 1, whereas non-mucosal healing as MES 2 or 3 throughout the colorectum. Furthermore, complete mucosal healing (cMH) was defined as MES 0. The evaluation of inflammation was performed by three physicians with endoscopic experience of 7 years or more. A majority vote was adopted during disagreements of opinions. Moreover, when endoscopic score was judged, clinical symptoms were blinded.

### SAA and CRP measurements

To measure SAA and CRP, blood specimens collected within 1 month before and after the colonoscopy were placed in a blood collection tube for biochemistry. SAA and CRP values were measured via an automatic analyzer using a latex agglutination reaction. The measurement kit for CRP was LZ Test Eiken CRP - HG and that for SAA was LZ Test Eiken SAA (Eikenkagaku, Tokyo, Japan). The measurement range was from 0.01 to 30 mg/dL for CRP and from 5 to 500 μg/mL for SAA. Both CRP and SAA used BM6070 (JEOL, Tokyo, Japan) as measuring equipment. In our hospital, when the SAA value was less than 5.6, the examination value was displayed as “< 5.6.” If the test result was less than 5.6, the value was set to 5.5, and statistical analysis was performed.

### Statistical analysis

All statistical analyzes were performed using EZR (Saitama Medical Center, Jichi Medical Center), a graphical user interface for R (The R Foundation for Statistical Computing, version 2.13.0) [13]. Spearman’s rank correlation was used to analyze the correlation between the test result (SAA and CRP), MES, and endoscopic findings. MES and inspection values (SAA and CRP) were evaluated using the Cochran-Armitage trend test. A receiver’s operating characteristic (ROC) curve was drawn to measure the area under the ROC curve and set a cutoff value. To infer the mucosal condition based on SAA and CRP values, sensitivity, specificity, positive predictive value (PPV), negative predictive value (NPV), and accuracy with 95% confidence interval were calculated. A *p*-value < 0.05 was considered to indicate a statistically significant difference.

### Ethical statement

Our study protocol conformed to the ethical standards of the responsible committees on human experimentation (institutional and national) and with the Declaration of Helsinki in 1964 and later versions and was approved by the Institutional Review Board of Hiroshima University Hospital. In this study, we only used clinical information without invasion or intervention to the patient, and we disclosed information such as research implementation and purpose and secured participants’ opportunity to refuse participation through posters.

## Results

### Patient characteristics

The patients’ background is shown in Table 1. A total of 199 colonoscopies were performed in 108 UC patients who underwent blood tests for CRP and SAA (63 men, 45 women). The median age at the time of endoscopic examination was 44 (range, 12–72) years, and the median disease duration at endoscopic examination was 9 (0–38) years. The median CAI value was 0 (range, 0–4) points. The Montreal classification was used to define the lesion range. The following number of cases were obtained: E1, 38 (19.1%); E2, 47 (23.6%); and E3, 114 (57.3%). The median MES was 1 (range, 0–3). Even in clinical remission patients, 39.6% of patients had endoscopic activity. The median CRP was 0.04 (range, 0.02–1.09) mg/dL, and the median SAA was 5.5 (range, 5.5–90.1) μg/mL. For treatment, 89.4, 9.0, 35.7, and 12.1% of the patients were administered with mesalazine, corticosteroids, immunomodulators, and biologics, respectively.

**Table 1:** Patient characteristics

Total no. of patients	108
Sex: male/female	63/45
Total colonoscopy	199
Number of colonoscopies	
1	58
2	30
≧3	20
Age during colonoscopy: median (range)	44 (12–72) years
Disease duration during colonoscopy: median (range)	9 (0–38) years
Montreal classification (%)	
E1/E2/E3	38 (19.1)/47 (23.6)/114 (57.3)
Clinical score at colonoscopy: median (range)	
CAI	0 (0–4)
Mayo endoscopic score: median (range)	1 (0–3)
Blood examination: median (range)	
CRP	0.04 (0.02–1.09)
SAA	5.5 (5.5–90.1)
Therapies (%)	
Mesalamine	178 (89.4)
Corticosteroids	18 (9.0)
Immunomodulators	71 (35.7)
Biologics	24 (12.1)

*CAI* Clinical activity index, *CRP* C-reactive protein, *SAA* Serum amyloid A

### Correlation between SAA, CRP, and colon endoscopic findings

**Fig. 1:**
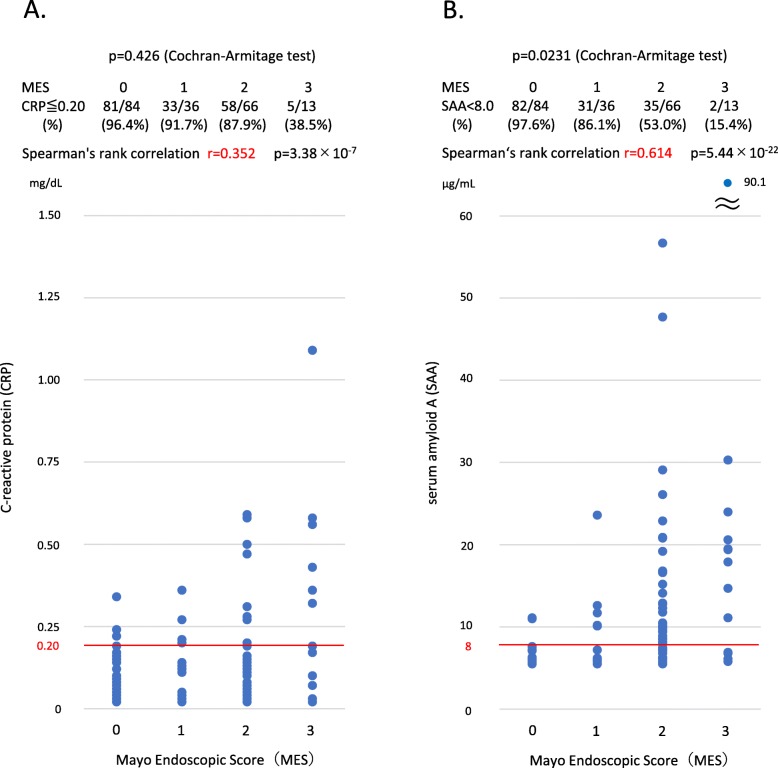
Correlation between CRP/SAA and endoscopic findings. Only a low correlation is found between CRP and MES (*r* = 0.352, *p* = 03.38 × 10^− 7^, **a**). On the contrary, SAA has a high correlation with MES (*r* = 0.614, *p* = 5.44 × 10^− 22^, **b**). Moreover, trends of MES and inspection values (SAA and CRP) were evaluated using the Cochran-Armitage trend test. At MES 0, the ratio at which CRP and SAA are normal is the highest, and with increasing MES, the ratio gradually decreases. The decreasing trend in relation to MES was statistically significant in SAA (*p* < 0.05), but not in CRP (**a**, **b**). CRP, C-reactive protein; MES, Mayo Endoscopic Score; SAA, serum amyloid A

We examined the correlation between SAA, CRP, and endoscopic findings. The results are shown in Fig. 1. A low correlation was found between CRP and MES (*r* = 0.352, *p* = 3.38 × 10^− 7^, Fig. 1a), whereas SAA had a high correlation with MES (*r* = 0.614, *p* = 5.44 × 10^− 22^, Fig. 1b). At MES 0, the ratio at which CRP and SAA are normal is the highest, and with increasing MES, the ratio gradually decreases. The decreasing trend in relation to the MES was statistically significant in SAA (*p* < 0.05), but not in CRP (Fig. 1a, b). The correlation between CRP, SAA, and MES was also examined in the same way, including in patients with no clinical remission (CAI > 5). Both CRP and SAA were highly correlated with MES, and SAA was more strongly associated with MES. CRP showed a stronger correlation than in clinical remission patients alone (data not shown).

### Diagnostic accuracy of SAA and CRP for mucosal healing

**Fig. 2:**
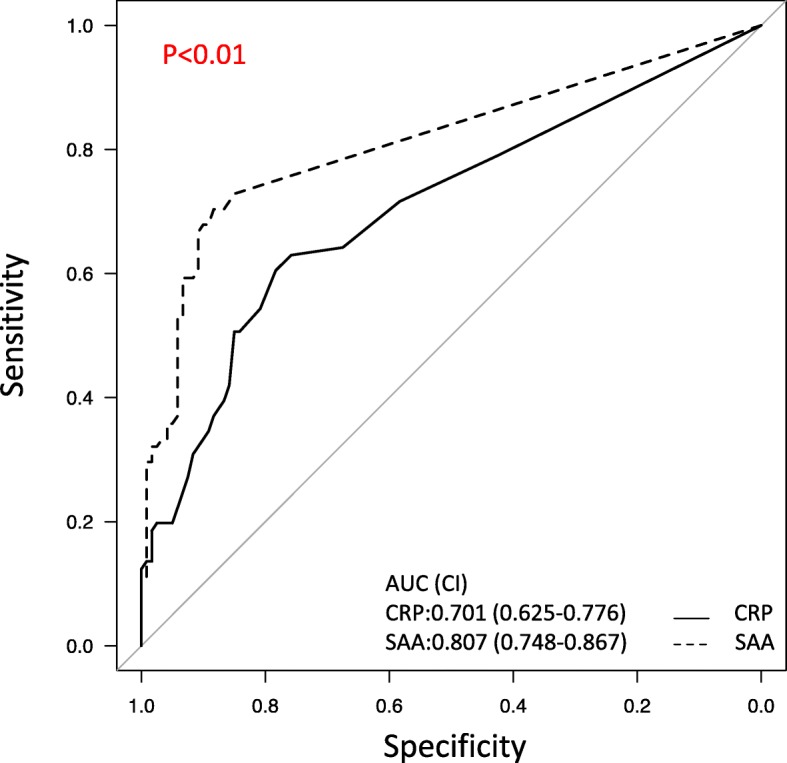
Comparison of ROC curves for mucosal inflammation (MES 2 or 3) of SAA and CRP. The ROC curves of SAA and CRP in patients in clinical remission. In the ROC analysis, the area under the ROC curve of the SAA was 0.807, indicating a higher predictive power, but that of CRP was 0.701. Comparison of ROC curves for mucosal inflammation (MES 2 or 3) of SAA and CRP showed that SAA was superior and indicated statistical significance (*p* < 0.01). AUC, area under the receiver’s operating characteristic curve, CI, confidence interval; CRP, C-reactive protein; MES, Mayo Endoscopic Score; SAA, serum amyloid A

Moreover, evaluation results of the predictive ability of SAA and CRP to identify mucosal inflammation in clinical remission patients are shown in Fig. 2. In the ROC analysis, the area under the ROC curve of the SAA was 0.807, indicating a higher predictive power, whereas that of the CRP was 0.701. Comparison of ROC curves for mucosal inflammation of SAA and CRP showed that SAA was superior to CRP and indicated a statistical significance (*p* < 0.01). Table 2 shows the sensitivity, specificity, PPV, NPV, and accuracy of CRP and SAA at optimal cutoff values for mucosal inflammation in clinical remission patients. SAA levels < 5.8 could discriminate mucosal inflammation from mucosal healing with sensitivity of 0.722, specificity of 0.850, PPV of 0.760, NPV of 0.823, and accuracy of 0.799. On the contrary, CRP levels < 0.060 could distinguish mucosal inflammation from mucosal healing with sensitivity of 0.620, specificity of 0.758, PPV of 0.628, NPV of 0.752, and accuracy of 0.704 (Table 2). The results indicate that SAA could be an excellent marker in predicting mucosal healing in clinical remission patients than CRP. We compared the area under the ROC curve of SAA and CRP for mucosal inflammation, including patients who did not reach clinical remission (CAI > 5). No significant difference was found (data not shown).

**Table 2:** Ability to predict mucosal inflammation (MES 2 or 3) with optimal cutoff value by ROC curve

	CRP	SAA
Sensitivity (95% CI)	0.620 (0.504–0.727)	0.722 (0.609–0.817)
Specificity (95% CI)	0.758 (0.672–0.832)	0.850 (0.773–0.909)
PPV (95% CI)	0.628 (0.511–0.735)	0.760 (0.647–0.851)
NPV (95% CI)	0.752 (0.665–0.826)	0.823 (0.744–0.885)
Accuracy (95% CI)	0.704 (0.635–0.766)	0.799 (0.737–0.852)

*CI* Confidence interval, *CRP* C-reactive protein, *MES* Mayo Endoscopic Score, *NPV* Negative predictive interval, *PPV* Positive predictive value, *SAA* Serum amyloid A

**Fig. 3:**
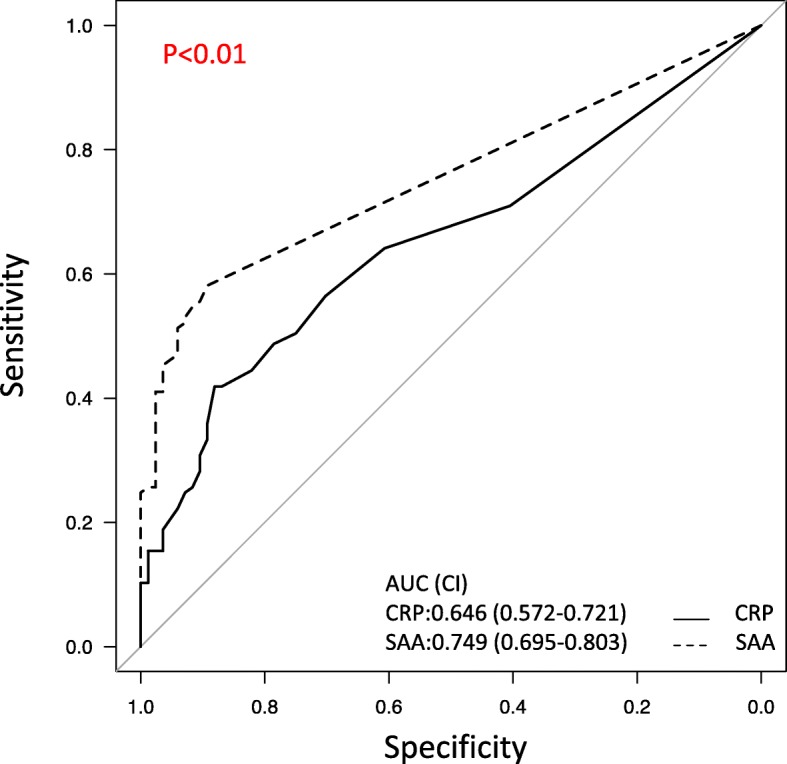
Comparison of ROC curves for mucosal inflammation (MES 1 or 2 or 3) of SAA and CRP. The ROC curves of SAA and CRP in patients in clinical remission. In the ROC analysis, the area under the ROC curve of the SAA was 0.749, indicating a higher predictive power, whereas that of the CRP was 0.646. Comparison of ROC curves for mucosal inflammation (MES 1 or 2 or 3) of SAA and CRP showed that SAA was superior and indicated statistical significance (*p* < 0.01). AUC, area under the receiver’s operating characteristic curve; CI, confidence interval; CRP, C-reactive protein; MES, Mayo Endoscopic Score; SAA, serum amyloid A

Recently, patients who achieved MES 0 have better prognosis compared to other patients [14, 15], and a higher treatment target is required for mucosal healing. Therefore, sensitivity and specificity, among others, were calculated by using mucosal healing only as MES 0 using the same method described above, and a ROC curve was drawn in clinical remission patients. The ROC, sensitivity, specificity, PPV, NPV, and accuracy with optimal cutoff values of SAA and CRP are shown in Fig. 3 and Table 3. Comparison of ROC curves for mucosal inflammation showed that SAA was superior than CRP, with statistically significant difference (*p* < 0.01), but its sensitivity was low.

**Table 3:** Ability to predict mucosal inflammation (MES 1 or 2 or 3) with optimal cutoff value by ROC curve

	CRP	SAA
Sensitivity (95% CI)	0.557 (0.461–0.649)	0.574 (0.478–0.666)
Specificity (95% CI)	0.702 (0.593–0.797)	0.893 (0.806–0.950)
PPV (95% CI)	0.719 (0.614–0.809)	0.880 (0.784–0.944)
NPV (95% CI)	0.536 (0.439–0.632)	0.605 (0.513–0.691)
Accuracy (95% CI)	0.618 (0.547–0.686)	0.709 (0.640–0.771)

*CI* Confidence interval, *CRP* C-reactive protein, *MES* Mayo Endoscopic Score, *NPV* Negative predictive interval, *PPV* Positive predictive value, *SAA* Serum amyloid A

**Fig. 4:**
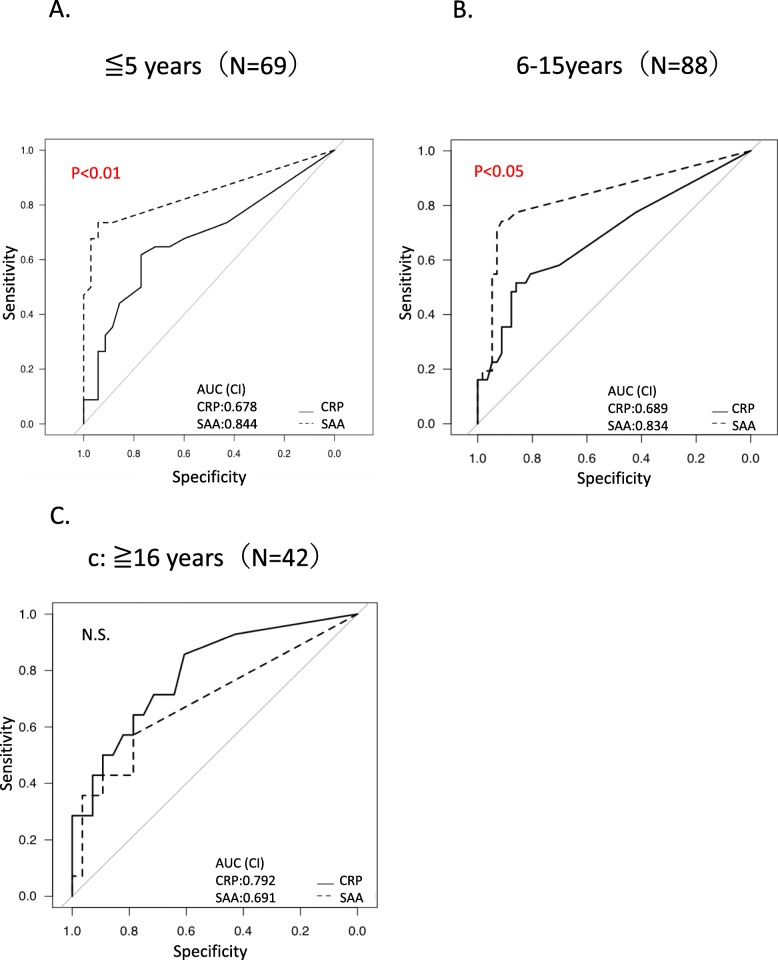
Comparison of ROC curves of mucosal inflammation (MES 2 or 3) of SAA and CRP when divided by disease duration. The ROC curves of SAA and CRP in patients in clinical remission when divided by disease duration. Disease duration was divided into three groups: 0–5 years (*N* = 69), 6–15 years (*N* = 88), and more than 16 years (*N* = 42). When examined by disease duration, comparison of ROC curves for mucosal inflammation (MES 2 or 3) of SAA and CRP showed that SAA was superior and indicated statistical significance in groups with disease duration of 0–5 years and 6–15 years. On the other hand, no statistical significance was shown in the group with disease duration of 16 years or more. AUC, area under the receiver’s operating characteristic curve; CRP, C-reactive protein; MES, Mayo Endoscopic Score; SAA, serum amyloid A

In addition, we examined by disease duration about patients in clinical remission. Disease duration was divided into three groups: 0–5 years (*N* = 69), 6–15 years (*N* = 88), and more than 16 years (*N* = 42). We examined the ability of SAA and CRP to predict mucosal inflammation by comparing the areas under the ROC curve. There was no difference between the three groups regarding disease type, age, and CAI (data not shown). In the groups with disease duration of 0–5 years and 6–15 years, SAA was significantly better than CRP, but in the group with disease duration of 16 years or more, the area under the ROC curve of CRP was high and SAA was not significant (Fig. 4).

We also examined disease type. Patients in clinical remission were divided into two groups: proctitis or left colitis (E1 + E2) and total colitis (E3). The area under the ROC curve of SAA and CRP for predicting mucosal inflammation in each group was compared. There was a significant difference in the E3 group, but there was no significant difference in the E1 + E2 group (data not shown). There was no difference between the two groups regarding age, disease duration, and CAI (data not shown). Therefore, the usefulness of SAA may be more enhanced in the group with widespread inflammation such as total colitis.

## Discussion

In this study, we examined whether SAA better predicts mucosal healing in UC patients in clinical remission compared to CRP. Our findings revealed that SAA has a strong correlation with endoscopic findings and is an excellent serum biomarker for predicting endoscopic activity in this patient cohort.

Monitoring of disease activity in routine practice is an important aspect in the clinical management of UC patients. It is very important to periodically examine clinical symptoms and endoscopic findings in such a population to determine the state of the colonic mucosa. However, frequent endoscopic examinations are difficult to perform; hence, biomarkers reflecting endoscopic findings are important.

Recently, fecal calprotectin has been used to evaluate mucosal inflammation, and its effectiveness has also been reported. Measuring fecal calprotectin levels has been proposed as a noninvasive test for evaluation of intestinal inflammation in IBD patients [16, 17]. However, because of the complexity of collecting feces, the lack of the result on the same day in some hospitals, and fluctuating values even when measured on the same day, fecal calprotectin might not remain clinically useful [18].

Therefore, we considered that SAA used as an inflammatory marker might predict mucosal healing in UC. CRP and SAA are secreted mainly by hepatocytes produced in response to infection, trauma, and other inflammatory conditions [6, 7]. These serum concentrations increase sharply and slowly return to normal levels over several days. However, chronic inflammation causes a sustained increase of these serum concentrations [6, 7]. Although there is a positive correlation between CRP and SAA concentrations [8, 19], studies have shown that SAA can be a more sensitive marker of inflammation in certain diseases, such as rheumatoid arthritis, primary biliary cirrhosis, and chronic active hepatitis [8]. Therefore, we decided to evaluate whether SAA measurement is a better serum biomarker than CRP. A recently published study revealed that SAA correlates with endoscopic findings in patients with CD and that SAA can be a useful biomarker to predict mucosal healing [20]. On the other hand, there is no report examining the correlation between UC and endoscopic findings yet. Hence, this report is the first to describe the correlation between SAA and intestinal mucosal inflammation. In UC patients, there was a positive correlation between mucosal inflammation and SAA, with the correlation being stronger than that of CRP, and SAA was found to more accurately reflect the state of the mucosa. In comparison with CRP, SAA proved to be an excellent marker for predicting mucosal inflammation in clinical remission patients. Although the therapeutic goal of UC is mucosal healing, clinical and endoscopic findings do not necessarily match. Actually, even in this study, 39.6% of clinical remission patients did not achieve mucosal healing. Therefore, among the clinical remission patients without symptoms, it is clinically important to evaluate intestinal inflammation using biomarkers than through frequent endoscopies. Although fecal markers, such as calprotectin, are considered useful, there are also limitations, as described above. Moreover, although CRP is still the most widely used serum biomarker, the existence of serum biomarkers that can more accurately predict mucosal healing is ideal.

Endoscopic examinations should be considered in clinical remission patients with elevated SAA, even if the CRP results are negative. Moreover, considering that measuring SAA is inexpensive and that the results are known on the same day, more facilities can adopt this approach and the financial burden on patients can be reduced. Excellent clinical outcome of patients with UC showing cMH (defined as MES 0) [15] has been set as the clinical goal in the treatment and management of these patients. SAA is an excellent marker for predicting mucosal inflammation than CRP. However, its diagnostic rate is lower when mucosal healing is defined as MES 0 or 1, indicating that SAA is an excellent marker in predicting strong mucosal inflammation only to some extent. We also compared the area under the ROC curve of SAA and CRP to predict mucosal inflammation in all patients, including those who did not reach clinical remission (CAI < 5). No significant difference was found. When the disease activity of UC increases, CRP level also tends to increase, and the significance of SAA decreases. Thus, SAA can be a better monitoring tool to predict mucosal inflammation than CRP in patients with clinical remission with low disease activity.

SAA is produced by the liver; it has recently been reported that it is also produced extrahepatically (Intestinal epithelium) [21]. In this study, SAA was not superior to CRP as a marker to predict mucosal inflammation when disease duration was prolonged. Longer disease duration may make it difficult for SAA to develop due to scarring and mucosal atrophy.

This study has several limitations. First, it is a retrospective study at a single facility involving a small absolute number of patients with UC. Excluding patients who were administered new therapies during the period from colonoscopy to the time CRP and SAA measurements were taken and those who were not in clinical remission may also be a selection bias. In addition, although no new treatment has been introduced in the included patients, the patients’ condition may have slightly changed because the date of the endoscopic examination and the date of the blood test are different. Second, since not all patients underwent urine tests, chest X-ray examination, computed tomography, etc., we cannot completely exclude infectious diseases and malignant tumors that may have caused the elevated CRP and SAA levels. Further prospective studies that are able to address these problems are needed. Third, we did not compare SAA with fecal markers, such as calprotectin. Calprotectin, in spite of its limitations as we mentioned above, is the most well established marker of mucosal disease at present. A future study comparing the serum markers with fecal markers could be interesting. The strengths of the study were as follows: it demonstrated the correlation between endoscopic findings of UC and SAA, and because it is a blood test, which can be easily measured, we believe that it can be applied immediately in the clinical setting.

## Conclusions

In conclusion, SAA has a strong correlation with endoscopic findings and is an excellent marker than CRP for predicting endoscopic activity in UC patients in clinical remission.

## Abbreviations

CAI: Clinical activity index
CD: Crohn’s disease
cMH: complete mucosal healing
CRP: C-reactive protein
IBD: Inflammatory bowel disease
NPV: Negative predictive value
PPV: Positive predictive value
ROC: receiver’s operating characteristic
SAA: Serum amyloid A protein
MES: Mayo endoscopic subscore
